# *Paenibacillus hubeiensis* sp. nov.: A Novel Selenium-Resistant Bacterium Isolated from the Rhizosphere of *Galinsoga parviflora* in a Selenium-Rich Region of Enshi, Hubei Province

**DOI:** 10.3390/microorganisms13071559

**Published:** 2025-07-02

**Authors:** Jiejie Kong, Ziyue Fu, Yueyang Liu, Can Jin, Xiaobo Peng, Xiaolong Liu, Yang Gao, Qiusheng Xiao, Yuting Su, Zhigang Zhao, Yunqiong Song, Xingjie Li, Daofeng Zhang

**Affiliations:** 1 Engineering Technology Research Center of Jiangxi Universities and Colleges for Selenium Agriculture, College of Life Science and Resources and Environment, Yichun University, Yichun 336000, China; kjj5autumn@163.com (J.K.); 17552644660@163.com (Y.L.); j2686099673@163.com (C.J.); pxb1276947507@163.com (X.P.); lxl032202@163.com (X.L.); gyyichuan@163.com (Y.G.); xqsycu@163.com (Q.X.); zhaozg_77@163.com (Z.Z.); 2 Institute of Marine Biology, College of Oceanography, Hohai University, Nanjing 210024, China; 211311040039@hhu.edu.cn; 3 Shanghai Agricultural Product Quality and Safety Center, Shanghai 201708, China; songyunqiong_1989@163.com

**Keywords:** *Paenibacillus hubeiensis* sp. nov., polyphasic analysis, bac120 tree

## Abstract

ES5-4^T^, a Gram-positive, motile, aerobic, and rod-shaped strain, was isolated from the rhizosphere of *Galinsoga parviflora* growing in the selenium-rich ore area of Enshi, Hubei Province, China. This strain can grow at pH levels of 5.0–10.0 and temperatures of 4–42 °C, with optimal growth at pH 7.0 and 28 °C. It was found to resist NaCl up to 5% (*w*/*v*), with an optimal growth condition of 0.5–1.0%. The strain exhibited tolerance to selenite (Se^4+^) concentrations up to 5000 mg/L. The major fatty acids of the ES5-4^T^ strain were anteiso-C_15:0_ (46.5%) and C_16:0_ (21.7%), its predominant respiratory quinone was MK-7, and its polar lipids included diphosphatidylglycerol (DPG), phosphatidylethanolamine (PE), phosphatidylglycerol (PG), and an unidentified phospholipid (PL). The presence of the 16S rRNA gene sequence implies that ES5-4^T^ belongs to a member of the genus *Paenibacillus*, with the highest sequence similarity of 98.4% to *Paenibacillus pabuli* NBRC 13638^T^. The bac120 tree also confirmed that the strain is within the genus *Paenibacillus*. The average nucleotide identity (ANI) and digital DNA–DNA hybridization (dDDH) values between ES5-4^T^ and closely related members of the genus *Paenibacillus* were all below the cutoff levels of 95–96% and 70%, respectively. Based on a polyphasic approach, including phenotypic, chemotaxonomic, and phylogenetic analyses, the ES5-4^T^ strain is proposed as a novel species of the genus *Paenibacillus*, for which the name *Paenibacillus hubeiensis* sp. nov. is proposed. This type strain is designated as ES5-4^T^ (=GDMCC 1.3540^T^ = KCTC 43478^T^).

## 1. Introduction

The genus *Paenibacillus* was originally described by Ash et al. based on 16S rRNA gene analysis [1] and later emended by Shida et al., who proposed six new species in the genus [2]. The continuous emergence of new members increased the number of species belonging to *Paenibacillus*. At the time of writing (June 2025), the genus contains 323 species with validly published and correct names (https://lpsn.dsmz.de/genus/paenibacillus, accessed on 29 June 2025). Members of this genus can be rod-shaped, aerobic or facultative anaerobic, motile or nonmotile, or endospore-forming bacteria [3,4,5].

*Paenibacillus* species can be found in many ecological niches, such as fish guts [6], river otters [7], seashore lands [8], hot springs [9], salt lakes [10], digestive syrups [11], plants [12], and soils [13]. Groups of species in this genus play a vital role in agricultural applications through nitrogen fixation, phosphate solubilization, the production of ACC deaminase, and IAA secretion [14,15,16] and are regarded as plant growth-promoting bacteria/rhizobacteria (PGPB/PGPR) [17,18,19].

Selenium (Se), an essential trace nutrient, has multiple biological functions, including roles in male reproductive health, muscle function, and the endocrine, central nervous, cardiovascular, and immune systems [20]. Research on Se has attracted extensive focus, partly because of selenocysteine, the constitutive part of the 21st amino acid, making it the only trace nutrient that is incorporated into proteins through genetic coding [21]. For example, certain Se functional microorganisms, called Se-respiring bacteria, play a pivotal role in the selenium cycle. Some selenate- or selenite-respiring bacteria are capable of producing Se nanoparticles [22]. Lin et al. [23] reported that *Priestia* sp. LWS1, a Se-resistant PGPR, promoted plant growth in *Oryza sativa* by 19% and increased Se accumulation by 75% compared with uninoculated treatment.

Therefore, screening and isolating agriculturally beneficial Se-resistant microorganisms from various habitats is imperative. In this study, a selenium-resistant PGPR known as ES5-4^T^ was isolated from the rhizosphere of *Galinsoga parviflora* and characterized using polyphasic analysis, with the results indicating that it is likely a novel species of the genus *Paenibacillus*.

## 2. Materials and Methods

### 2.1. Sample Collection and Isolation of Bacterial Strain

*G. parviflora* is among the dominant plant species found in the selenium-rich ore area of Enshi, Hubei Province, China (30°8′43″ N 109°49′32″ E), which is why the rhizobacterial soil was collected for this study in October 2020. The total selenium (Se) content of the soil is 3.57 mg/kg, which is higher than the average value of 0.4 mg/kg, thus making it a selenium-rich soil based on China’s Soil Selenium Content Grade (GB/T 44971-2024) [24]. The determination of Se content in soil was carried out according to the China’s Determination of Selenium in Soils (NY/T 1104-2006) [25]. Bacterial isolation was based on the method described by Li et al. [26]. Briefly, the soil sample was diluted to 10^−3^ to 10^−5^ with sterile ddH_2_O using a 10-fold dilution gradient and then smeared on a Trypticase soy broth agar (TSA; 15.0 g/L tryptone, 5.0 g/L peptone, 5.0 g/L NaCl, 15–20 g/L agar) medium supplemented with 20 mg/L of Se solution (Na_2_SeO_3_). After incubation for 3 days at 28 °C in an incubator, the single colony was selected, purified, and incubated on TSA plates with higher, successive Se concentrations (50, 100, 200, 500, 1000, and 2000 mg/L). Finally, the ES5-4^T^ strain was obtained and maintained on TSA and preserved in a 30% glycerol suspension at −80 °C.

### 2.2. 16S rRNA Gene Sequencing and Phylogenetic Analysis

The strain was cultivated in a Trypticase soy broth (TSB: 15.0 g/L tryptone, 5.0 g/L peptone, 5.0 g/L NaCl) medium for 12 h until it reached the exponential growth stage at 180 rpm in a rotary shaker (ZWY-2101C, Zhicheng, Shanghai, China) at 28 °C. Next, the cell suspensions were used for the extraction of genomic DNA using a DNA isolation kit (Sangon Biotech, Shanghai, China), according to the manufacturer’s instructions. The 16S rRNA gene was amplified by PCR using the universal primers 27F and 1492R [27], and the PCR products were purified and sequenced by Sangon Biotech. The obtained 16S rRNA gene sequence was analyzed using the EzBioCloud server (https://www.ezbiocloud.net/, accessed on 29 June 2025) [28]. Multiple sequence alignments were performed using the ClustalW software v 1.81 (http://www.clustal.org/clustal2/, accessed on 29 June 2025) [29]. Next, phylogenetic analyses of the 16S rRNA gene of the ES5-4^T^ strain were conducted based on neighbor-joining (NJ), maximum-parsimony (MP), and maximum likelihood (ML) algorithms using the MEGA 7 software v7.0 [30]. Phylogenetic distances were calculated according to Kimura’s two-parameter model [31], and the bootstrap values were set as 1000 replications, as described by Felsenstein [32].

### 2.3. Genomic Characterization

The ES5-4^T^ strain was incubated until it reached the exponential growth stage as mentioned above. The cell suspension was then centrifuged at 4 °C at 8000× *g*, the supernatant was discarded, and the cell was rinsed three times with sterile deionized water and collected and used for genome sequencing. A draft genome sequence of the ES5-4^T^ strain was generated using the Illumina HiSeq X platform (Personalbio, Shanghai, China). Raw sequence reads were filtered and quality controlled using FastQC [33]. The high-quality reads were de novo assembled using SPAdes v3.15.3 [34] in the UGENE software package v1.21 [35]. The G + C content of the genomic DNA was directly determined using the draft genome sequence. Values of average nucleotide identity (ANI) using BLAST (OrthoANIb) v1.40 between the ES5-4^T^ strain and its close relatives (*Paenibacillus illinoisensis* NBRC 15959^T^, *Paenibacillus pabuli* NBRC 13638^T^, *Paenibacillus taichungensis* DSM 19942^T^, and *Paenibacillus xylanivorans* A59^T^) were calculated on the EzBioCloud server [28]. The online Genome-to-Genome Distance Calculator (GGDC) (http://ggdc.dsmz.de/distcalc2.php, accessed on 29 June 2025) was used to calculate the digital DNA–DNA hybridization (dDDH) values by applying Formula 2 [36]. Annotation was performed using the NCBI Prokaryotic Genome Annotation Pipeline [37] and the online eggnog-mapper v2 server (http://eggnog-mapper.embl.de, accessed on 29 June 2025) [38].

To confirm the taxonomic status of the type strain, a genome-based ML phylogenetic tree (bac120 tree) [39] was constructed based on the protein sequences of the genus *Paenibacillus*, along with an outgroup. Generally, the genomic information of type strains in the genus *Paenibacillus* was collected using scripts (https://github.com/zdf1987/Usefull_Scripts/, accessed on 29 June 2025), and the proteomes were downloaded using the NCBI software v16+ datasets (https://www.ncbi.nlm.nih.gov/datasets/docs/v2/download-and-install/, accessed on 29 June 2025). Other genomic datasets of strains that are closely related to the ES5-4^T^ strain were downloaded manually. The gene sequences of each gene cluster were aligned using MUSCLE [40], and the ML tree was constructed using FastTree 2.1 [41] based on the common genes in the bac120 set after alignment trimming and concatenation. The bac120 tree was constructed automatically using EasyCGTree v4.2 (https://github.com/zdf1987/EasyCGTree4, accessed on 29 June 2025) [42].

### 2.4. Phenotypic and Biochemical Characterization

The phenotypic characteristics of the ES5-4^T^ strain were evaluated on TSA. Gram staining was conducted using a Gram-staining kit (DM0015, Leagene, Beijing, China). The strain’s cell morphology was visualized using scanning (Sirion 200) and transmission electron microscopes (SEM, FEI Sirion 200, Hillsboro, OR, USA; TEM, FEI Tecnai G2 spirit Bio-Twin, Hillsboro, OR, USA) [43]. SEM imaging was conducted at 10.0 kV with magnifications of 8000–30,000×, while TEM analysis employed 150 kV at 4000–20,000×. Representative micrographs were acquired with selected fields. Scale bars were annotated in all images. The effect of different temperatures (4, 10, 20, 28, 37, and 45 °C) on bacterial growth was observed on the TSA after 3 days of incubation. pH levels in the range of 4.0 to 10.0, adjusted with 1 M HCl or NaOH, with an increment of 1.0 for bacterial growth, were also assessed. Salt tolerance was evaluated using various NaCl concentrations (0%, 0.5%, 1.0%, 2.0%, 3.0%, 5%, 7%, and 10% *w*/*v*). The bacterial motility experiment was conducted by observing cell growth in test tubes with semisolid TSB with 0.5% agar after 3 days of incubation in an incubator at 28 °C. Other physiological and biochemical characteristics of the strain were determined using API 50CH (bioMérieux, Craponne, France) and Biology GEN III micro test systems (Biology, Boston, MA, USA). The reference strains (*Paenibacillus oceanisediminis* JCM17814^T^ and *Paenibacillus dongdonensis* KCTC 33221^T^) used in the study were provided by the Marine Culture Collection of China (MCCC). All experiments were performed in triplicate.

### 2.5. Chemotaxonomic Analysis

The ES5-4^T^ strain and its reference type strains were cultivated in TSB until the logarithmic phase was reached at 180 rpm in the rotary shaker (ZWY-2101C) at 28 °C. The cells were collected, washed, and lyophilized in a freeze dryer (Labconco, Kansas City, MO, USA) [44]. The dried cells were used for cellular fatty acid, respiratory quinone, and polar lipid analyses: cellular fatty acids were extracted and analyzed using the Sherlock Microbial Identification System (MIDI) following Sasser’s protocol [45]; quinones were purified and analyzed using high-performance liquid chromatography [46]; and polar lipids were first extracted based on the method of Minnikin et al. [47] and identified using two-dimensional thin-layer chromatography, according to the method of Komagata and Suzuki [48], in which molybdenum blue and phosphomolybdate reagents were also used.

### 2.6. Effects of Se^4+^ Concentration on the Growth of the ES5-4^T^ Strain

To assess the effect of Se^4+^ (used in the form of Na_2_SeO_3_) on the cell growth of the ES5-4^T^ strain, TSA and TSB media with various Se concentrations (0, 20, 50, 100, 200, 500, 1000, 2000, and 5000 mg/L) were prepared. For the TSB test, 600 μL of the overnight cultured cell suspension, equivalent to a 3% inoculation volume (*v*/*v*), was added to 50 mL sterile Erlenmeyer flasks containing 20 mL of TSB with the aforementioned Se^4+^ concentration and cultured for 2 days in the rotary shaker (ZWY-2101C) at 180 rpm at 28 °C. Next, the optical density at the 600 nm wavelength (OD_600_) was measured using a Multiskan Go microplate spectrophotometer (Thermo Fisher Scientific, Waltham, CA, USA). Cell growth on the TSA plates containing equivalent Se^4+^ concentrations was also assessed. Each plate was evenly divided into three compartments, where 0.5 μL of the overnight cultured cell suspension was dripped into each compartment and cultured in a biochemical incubator (Xinmiao, Shanghai, China) for 2 days at 28 °C, and the growth of the ES5-4^T^ strain was recorded. The diameter of the colonies of the ES5-4^T^ strain was measured using a digital vernier caliper (DWGR-2954, DELIXI, Shanghai, China). All experiments were performed in triplicate.

### 2.7. Identification of the Functional Gene for Selenocompound Metabolism

Twenty-two genes involved in selenocompound metabolism (map00450) in bacteria of the genus *Paenibacillus* were identified according to a strategy similar to that previously described [49]. Briefly, profile hidden Markov models (HMMs) of the 22 relevant genes were retrieved from the online KOfam database (https://www.genome.jp/ftp/db/kofam/, accessed on 29 June 2025) [50]. These HMMs were merged and adjusted to meet the requirements of the HMMER v3.0 software (http://hmmer.org, accessed on 29 June 2025), which was used by the analysis pipeline based on EasyCGTree v4.2 (https://github.com/zdf1987/EasyCGTree4, accessed on 29 June 2025) [42]. Subsequently, the “Gene_Prevelence.pl” script within the EasyCGTree software v4.2 package was used to search for homologous genomes using customized HMMs with an e-value cutoff of 1 × 10^−80^. All candidate genes were subsequently submitted to the online annotation tool KofamKOALA (https://www.genome.jp/tools/kofamkoala/, accessed on 29 June 2025) [50] for homology verification. Finally, the online tool ImageGP2 (https://www.bic.ac.cn/BIC/#/, accessed on 29 June 2025) was employed to generate a heatmap of the gene distribution.

### 2.8. Statistical Analysis

Data were analyzed using a one-way ANOVA. Pairwise differences among treatments were evaluated using Duncan’s multiple range test. Data were visualized using SigmaPlot 14.0 (Systat Software, San Jose, CA, USA). All statistical analyses were conducted using SPSS 20.0 (IBM, Armonk, NY, USA), with a significance level set at *p* < 0.05.

## 3. Results and Discussion

### 3.1. Phylogenetic Analysis of the 16S rRNA Gene

The 16S rRNA gene sequence of the ES5-4^T^ strain was 1403 bp in length, with three mismatches spanning the complete gene sequence (1552 bp; RefSeq accession NZ_JAVYAE020000044), and was deposited in the GenBank/EMBL/DDBJ databases (accession number: OR584159). The ES5-4^T^ strain shared the highest 16S rRNA gene sequence similarity (98.4%) with *P. pabuli* NBRC 13638^T^ based on the results from the EzBioCloud server [28], followed by *P. dongdonensis* KUDC 0114^T^ (98.3%) [51], *P. taichungensis* BCRC 17757^T^ (98.3%) [52], and *P. xylanivorans* A59^T^ (98.0%) [53]. Alignment of the full-length 16S rRNA gene against the genome database (refseq_genomes and wgs) on NCBI suggested that there were some genomes from uncharacterized bacteria sharing 16S rRNA gene identities of >98.5% with the ES5-4^T^ strain, including *Paenibacillus* sp. FSL W8-0426 (100%, NZ_CP150203.1), *P. pabuli* E1 (98.8%, NZ_CP073714.1), *P. xylanilyticus* W4 (98.7%, NZ_CP044310.1), *Paenibacillus* sp. 11B (98.6%, NZ JAUKNS010000005.1), *P. xylanilyticus* L-2 (98.6%, NZ CP182582.1), *Paenibacillus* sp. 1781tsa1 (98.58%, NZ JAMYWY010000001.1), *Paenibacillus* sp. PvP094 (98.5%, NZ JBEPOM010000001.1), and *Paenibacillus* sp. FSL R5-0713 (98.5%, NZ_CP150229.1).

**Figure 1:**
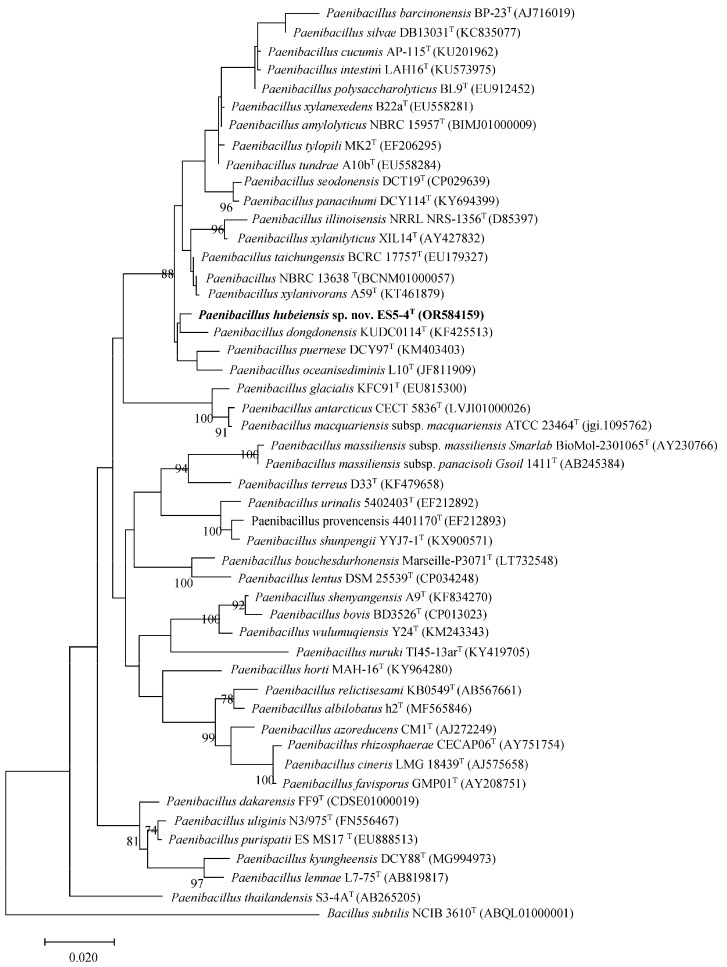
Maximum likelihood phylogenetic tree based on 16S rRNA gene sequences showing the position of the ES5-4^T^ strain. Bootstrap values (expressed as percentages of 1000 replications) of >70 (%) are shown at the branch nodes. GenBank accession numbers are indicated in brackets at the end of the tip labels. *Bacillus subtilis* NCIB 3610^T^ is used as the outgroup. Bar = 0.02 substitutions per nucleotide position.

Phylogenetic analysis using the ML method indicated that the ES5-4^T^ strain clustered with *P. dongdonensis* KUDC 0114^T^ (Figure 1), while the MP method revealed that the strain clustered with *P. illinoisensis* NRRL NRS-1356^T^ and *P. xylanilyticus* XIL 14^T^ (Figure S1). The above two topologies could not be reproduced in the trees using the NJ method (Figure S2). In addition, this phylogeny was not well supported in all three trees (bootstrap value < 70%). These observations suggested that phylogenetic inference based solely on the 16S rRNA gene may have limited resolution within this genus. *P. dongdonensis* KUDC 0114^T^ (=KCTC 33221^T^) and *P. oceanisediminis* L 10^T^ (=JCM 17814^T^) were used as reference strains in the subsequent tests because they have no available genomes to distinguish themselves from the ES5-4^T^ strain on a genome basis.

### 3.2. Genome-Based Characteristics

The results of the Whole-Genome Shotgun project of the ES5-4^T^ strain were deposited in the GenBank/EMBL/DDBJ databases with the accession number JAVYAE000000000. The draft genome contained 58 contigs, with a total length of 7,190,355 bp, and the genome G + C content was 50.5%. A total of 6300 protein-coding genes were predicted, of which 642, 3046, and 5370 genes were assigned to the Gene Ontology (GO), Kyoto Encyclopedia of Genes and Genomes (KEGG), and Cluster of Orthologous Groups (COG) databases, respectively. Further KEGG pathway analysis revealed 34 genes involved in flagellar assembly (ko02040), 3 genes (*narI*, *narJ*, and *narH*) associated with nitrate reduction, 3 genes (*cysI*, *cysJ*, and *nirB*) associated with sulfite reduction, the nitrogen fixation gene *nifU*, and 317 ABC transporters (ko00920, ko01501, ko01503, ko02010, ko02020, ko02024, ko05150, ko05152), while no photosynthesis gene cluster was detected.

**Figure 2:**
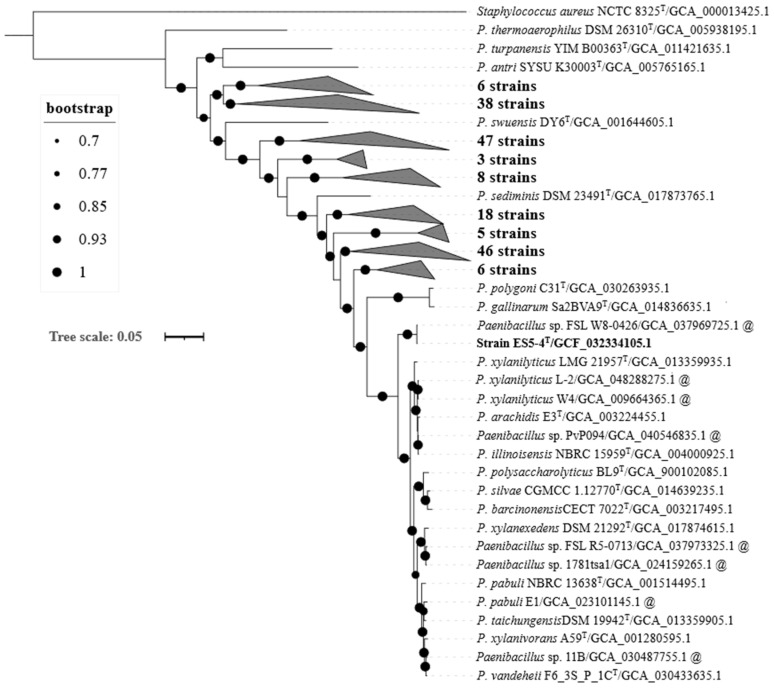
A maximum likelihood tree based on protein sequences of the bac120 gene set, showing the phylogenetic relationship of ES5-4^T^ in the genus *Paenibacillus*. Bootstrap values are displayed at the branch nodes. The GenBank assembly accession number is indicated in brackets. The @ symbol at the end of labels indicates an atypical strain that showed 16S rRNA gene similarities of >98.5%. *Staphylococcus aureus* NCTC 8325^T^ (GCA000013425.1) is used as an outgroup. Bar = 0.1 substitutions per amino acid position.

According to the taxa of the 16S rRNA gene tree, 205 *Paenibacillus* genomes (including those of the seven aforementioned atypical strains together with the genomes of the ES5-4^T^ strain and *Staphylococcus aureus* NCTC 8325^T^ (GCA000013425.1, as the outgroup)) were used to infer a robust tree based on the bac120 gene set (Figure 2). The bac120 tree was well supported on all branches. The ES5-4^T^ strain was closely related to *Paenibacillus* sp. FSL W8-0426 (GCA_037969725.1) and was subsequently clustered with ten *Paenibacillus* species, including *P. pabuli* NBRC 13638^T^, *P. taichungensis* DSM 19942^T^, and *P. xylanivorans* A59^T^. The results of the 16S rRNA gene tree and the bac120 tree both demonstrated that the strain was within the genus *Paenibacillus*.

The ANIb values between the ES5-4^T^ strain and its closely related strains of *P. pabuli* NBRC 13638^T^ (GCA004000925.1), *P. taichungensis* DSM 19942^T^ (GCA013359905.1), *P. xylanivorans* A59^T^ (GCA001280595.1), and *P. illinoisensis* NBRC 15959^T^ (GCA004000925.1) were 76.4%, 76.2%, 76.3%, and 76.8%, respectively, with corresponding dDDH values of 21.2%, 20.9%, 20.6%, and 21.6% (Table 1). These values were lower than the proposed and acceptable species boundaries of 95–96% for ANI and 70% for dDDH [54,55]. In addition, the ES5-4^T^ strain showed an ANI value of 98.8% with an unpublished and invalidly named *Paenibacillus* sp. FSL W8-0426 (GCA_037969725.1) strain, which had a 16S rRNA gene similarity of 100%. Taken together, these findings imply that the ES5-4^T^ strain represents a novel *Paenibacillus* species.

### 3.3. Phenotypic and Biochemical Characteristics

**Figure 3:**
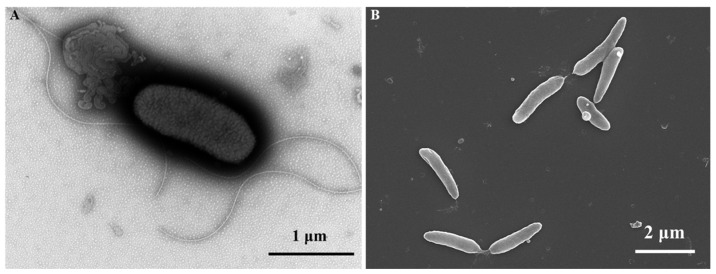
The electron microscopy images of the ES5-4^T^ strain. (**A**) The TEM images (15,000× magnification) showing the cell morphology of the ES5-4^T^ strain cultured on TSA media at 28 °C for 2 days. (**B**) The SEM images (10,000× magnification) showing the cell morphology of the ES5-4^T^ strain cultured on TSA media at 28 °C for 1 day.

The ES5-4^T^ strain was found to be Gram-positive, aerobic, and motile with flagella (Figure 3A), and these features were consistent with the description of *Paenibacillus flagellatus* [56]. The SEM images of the ES5-4^T^ cells showed a rod-shaped profile with a width of 0.4–0.6 μm and a length of 1.0–2.5 μm (Figure 3B). Colonies on TSA were white, circular, moist, and smooth after incubation for 72 h at 28 °C. Growth occurred at NaCl concentrations of 0–5.0% (*w*/*v*), pH levels of 5.0–10.0, and temperatures of 4–42 °C. The optimal growth conditions for the ES5-4^T^ strain were 0.5–1.0% (*w*/*v*) NaCl, pH levels of 5.0–7.0, and 28 °C; those for *P. dongdonensis* KCTC 33221^T^ were 0.5–2.0% (*w*/*v*) NaCl, pH levels of 6.0–7.0, and 28 °C; and those for *P. oceanisediminis* JCM17814^T^ were 0.5–1.0% (*w*/*v*) NaCl, pH levels of 7.0–8.0, and 28 °C (Table 2).

In the API 50CH tests, the ES5-4^T^ strain was only positive for esculin and negative for L-arabinose, ribose, D-xylose, β-methyl-D-xyloside, galactose, glucose, fructose, rhamnose, mannitol, amygdalin, arbutin, salicin, cellobiose, maltose, lactose, melibiose, saccharose, trehalose, raffinose, and gluconate, unlike reference strains *P. oceanisediminis* JCM17814^T^ and *P. dongdonensis* KCTC 33221^T^, which displayed positive reactions in these tests. Compared with the reference strain *P. dongdonensis* KCTC 33221^T^, which was positive for H_2_S production and the hydrolysis of starch, cellulose, and Tween 20, 40, and 60, the ES5-4^T^ strain was only positive for H_2_S production and the hydrolysis of Tween 40 and 60. *P. oceanisediminis* JCM17814^T^ was positive for the hydrolysis of starch, cellulose, and Tween 40 and negative for other relative tests (Table 2).

In the BIOLOG GEN III tests, the ES5-4^T^ strain was positive for α-D-glucose, D-mannose, D-fructose, D-galactose, D-fucose, L-glutamic acid, L-serine, D-galacturonic acid, L-galactonic acid lactone, D-gluconic acid, D-glucuronic acid, glucuronamide, mucic acid, quinic acid, citric acid, and L-malic acid. In comparison, *P. oceanisediminis* JCM17814^T^ was negative for the utilization of D-fucose, L-glutamic acid, L-serine, glucuronamide, mucic acid, and citric acid, whereas *P. dongdonensis* KCTC 33221^T^ was negative for L-serine and glucuronamide. These differential characteristics among the three strains are summarized in Table 2.

### 3.4. Effects of Se^4+^ Concentration on the Growth of the ES5-4^T^ Strain

**Figure 4:**
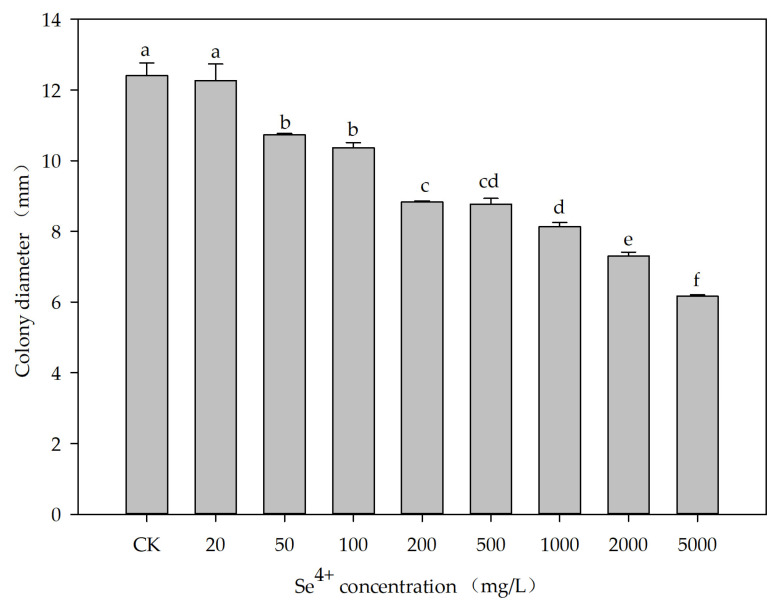
Effects of Se^4+^ concentration (20–5000 mg/L) on the colony diameter of the ES5-4^T^ strain cultured on TSA media at 28 °C for 2 days. Different letters above the column indicate significant differences between treatments (*p* < 0.05).

The effect of Se^4+^ concentration on the growth of the ES5-4^T^ strain was investigated using TSB and TSA media. As Se^4+^ concentration increased, the cell suspension color changed from yellow to deep orange-red and then to light orange-red in the TSB (Figure S3), indicating that the white Se^4+^ was converted to red selenium nanoparticles (SeNPs) by the ES5-4^T^ strain [57]. The optical density values at 600 nm (OD_600_) were disturbed by the red color caused by SeNPs. Therefore, the values showed a trend of first decreasing, then increasing, and then decreasing again, reaching their maximum at a Se^4+^ concentration of 500 mg/L (Figure S3). The cell colors changed from white to red when the ES5-4^T^ strain grew on TSA (Figure S4). In addition, with an increase in the Se^4+^ concentration, the colony diameter seemed to decrease significantly (*p* < 0.05) (Figure 4). *Bacillus* sp. KW3, *Corynebacterium* sp. VS5, and *Pseudomonas* sp. VW7 have been found to be resistant to Se^4+^ above 200 mM [58]. The minimal inhibitory concentrations of *Lysinibacillus xylanilyticus* and L. *macrolides* for Se^4+^ were 120 and 220 mM, respectively [59]. The ES5-4^T^ strain is resistant to Se^4+^ concentrations of at least 5000 mg/L, approximately corresponding to 30 mM.

In addition to the ES5-4^T^ strain, many other *Paenibacillus* species can also reduce white Se^4+^ to red SeNPs. For example, *Paenibacillus selenitireducens* ES3-24^T^, isolated from a selenium mineral soil, was able to reduce Se to red elemental selenium [60]. Long et al. [61] reported the same function of *Paenibacillus motobuensis* LY5201.

SeNPs are versatile in their applications, having antioxidant [62], antibacterial [63], anticancer, and immunomodulatory characteristics [64], exhibiting bioremediation [65], heavy metal detoxification, and nutraceutical functions, and playing a role in the fortification of dietary selenium, especially in selenium-deficient areas [66]. The ES5-4^T^ strain might also be applicable in the aforementioned aspects, but this requires further research.

### 3.5. Genetic Features of Paenibacillus for Selenocompound Metabolism

**Figure 5:**
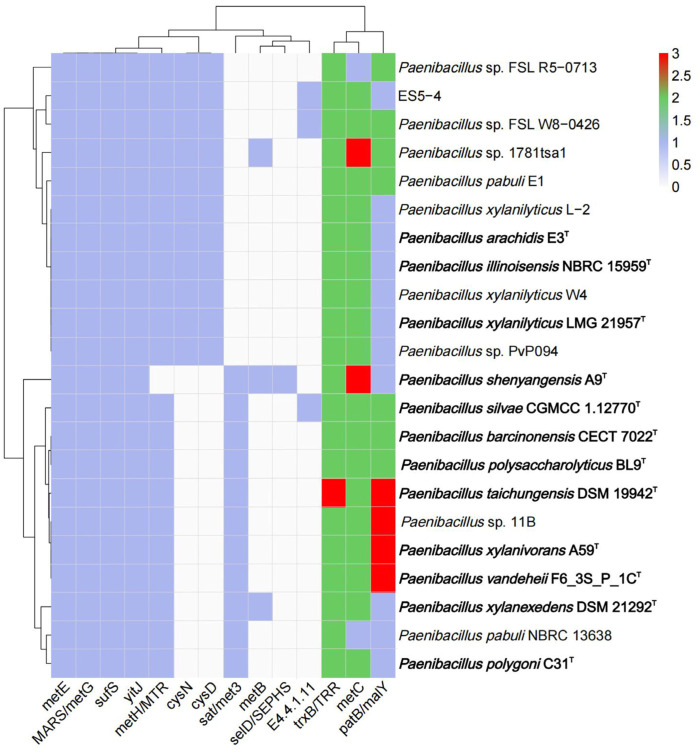
The distribution of genes for selenocompound metabolism within the genus *Paenibacillus*. Type strains are marked in bold. Gradient colors indicate gene numbers (0–3) detected in the relevant gene family.

Given the ES5-4^T^ strain’s resistance to Se^4+^, the distribution of the 22 genes involved in selenocompound metabolism was analyzed in the *Paenibacillus* genus, and 14 genes were detected (Figure 5; Dataset S1). *trxB/TRR*, *metC*, *patB/malY*, *metE*, *MARS/metG*, *sufS*, *yitJ*, and *metH/MTR* genes were almost universal in these strains, with *metH/MTR* being absent in *Paenibacillus shenyangensis* A9. *selD/SEPHS* was only present in *P. shenyangensis* A9, which catalyzes selenophosphate synthesis using inorganic selenium and ATP as substrates [67]. *metB*, which can catalyze selenocysteine to selenocystathionine [68], was only present in *P. xylanexedens* DSM 21292, *P. shenyangensis* A9, and *Paenibacillus* sp. 1781tsa1. The gene implicated in the E4.4.1.11 reaction, which produces methaneselenol from selenomethionine [69], was only found in *Paenibacillus* sp. FSL W8-0426, *P. silvae* CGMCC 1.12770, and the ES5-4^T^ strain (Figure 5). The thioredoxin reductase gene *trxB/TRR* catalyzed selenite (SeO_3_^2−^) to hydrogen selenide (H_2_Se), which was spontaneously oxidized by oxygen to become Se (0) and eventually formed SeNPs [70,71,72]. Cystathionine-β-lyase, coded by *metC*, catalyzes selenocystathionine to selenohomocysteine [73,74]. Together, the results suggest that species in the *Paenibacillus* genus are versatile in selenocompound metabolism. Notably, the ES5-4^T^ strain carries two copies of *metC* and *trxB/TRR*, which likely confer its selenium resistance (Figure 5).

### 3.6. Chemotaxonomic Characteristics

The major cellular fatty acids (>10%) were anteiso-C_15:0_ (46.5%) and C_16:0_ (21.7%) for the ES5-4^T^ strain; iso-C_14:0_ (10.1%), anteiso-C_15:0_ (45.6%), and iso-C_16:0_ (20.0%) for *P. oceanisediminis* JCM 17814^T^; and C_16:0_ (18.3%) and anteiso-C_15:0_ (50.3%) for *P. dongdonensis* KCTC 33221^T^ (Table S1). The polar lipids of the ES5-4^T^ strain mainly comprised diphosphatidylglycerol (DPG), phosphatidylethanolamine (PE), phosphatidylglycerol (PG), and an unidentified phospholipid (PL), which is similar to that of the closely related strain *P. oceanisediminis* JCM17814^T^ (Figure S5). The predominant respiratory quinone of the ES5-4^T^ strain was MK-7, which is in accordance with the *Paenibacillus* genus [75,76].

## 4. Conclusions

A strain designated as ES5-4^T^ was isolated from the rhizosphere (with a Se content of 3.57 mg/kg) of *G. parviflora* growing in the selenium-rich ore area of Enshi, Hubei Province, China. 16S rRNA and bac120 gene trees indicated that the ES5-4^T^ strain formed a distinct clade in the genus *Paenibacillus*. The ANI and dDDH values between ES5-4^T^ and its closely related species were below the boundaries of 95–96% for ANI values and of 70% for dDDH values. Furthermore, the ES5-4^T^ strain differed from the reference strains *P. oceanisediminis* JCM17814^T^ and *P. dongdonensis* KCTC 33221^T^ in phenotypic and chemotaxonomic characteristics, such as growth conditions, motility, starch and cellulose hydrolysis, the utilization of some substrates, and the presence of polar lipids and cellular fatty acids. Based on these phenotypic, chemotaxonomic, and genotypic features, the ES5-4^T^ strain can be considered a novel *Paenibacillus* species, for which the name *Paenibacillus hubeiensis* sp. nov. is proposed. To the best of our knowledge, this is the first report of a Se-tolerant *Paenibacillus* species associated with *G. parviflora* in a natural selenium-rich environment.

### Description of Paenibacillus hubeiensis *sp. nov.*

*Paenibacillus hubeiensis* (hu.bei.en’sis. N.L. masc. adj. *hubeiensis*, pertaining to the province of Hubei, where the selenium-rich ore is located, from which the strain was isolated).

The strain (ES5-4^T^) is Gram-positive, motile, aerobic, and rod-shaped. The colonies on TSA are milky, circular, moist, and smooth after incubation for 3 days at 28 °C. The cells can be grown at 4–42 °C (optimum, 28 °C) and pH levels of 5.0–10.0 (optimum, 5.0–7.0) and tolerates up to 5% (optimum, 0.5–1.0%) of NaCl. The strain can tolerate up to 5000 mg/L of Se^4+^ and can produce SeNPs. In the API 50CH analysis, it is positive only for esculin, which is in the context of 49 other substrates. In the BIOLOG GEN III tests, it is positive for 16 types of organic carbon sources. The major fatty acids are anteiso-C_15:0_ (46.5%) and C_16:0_ (21.7%), the predominant respiratory quinone is MK-7, and the polar lipids mainly comprise diphosphatidylglycerol (DPG), phosphatidylethanolamine (PE), phosphatidylglycerol (PG), and an unidentified phospholipid (PL).

The type strain, ES5-4^T^ (=GDMCC 1.3540^T^ = KCTC 43478^T^), was isolated from the rhizosphere of *G. parviflora*, which was collected from a selenium-rich ore area of Enshi, Hubei Province, China (30°8′43″ N 109°49′32″ E). The genomic G + C content of the strain was 50.5%. The 16S rRNA gene sequence and genome were deposited in the GenBank/EMBL/DDBJ databases with accession numbers OR584159 and JAVYAE000000000, respectively.

## Figures and Tables

**Table 1:** Average nucleotide identity based on BLAST (OrthoANIb) and digital DNA–DNA hybridization (dDDH) analysis for the ES5-4^T^ strain and its closely related species.

Strain	1	2	3	4	5
1	-	76.4	76.2	76.3	76.8
2	21.2	-	88.6	88.4	80.5
3	20.9	36.3	-	91.8	80.4
4	20.6	35.9	45.6	-	80.5
5	21.6	23.8	29.9	23.6	-

Note: 1, strain ES5-4^T^; 2, *Paenibacillu pabuli* NBRC 13638^T^; 3, *Paenibacillu taichungensis* DSM 19942^T^; 4, *Paenibacillu xylanivorans* A59^T^; 5, *Paenibacillu illinoisensis* NBRC 15959^T^. OrthoANIb results are shown above the diagonal, and dDDH results are shown below the diagonal. The values are in percentages.

**Table 2:** Differential characteristics between the ES5-4^T^ strain and its closely related species.

Characteristic	1	2	3
Motile	+	−	−
Optimal temperature (°C)	28	28	28
Optimal NaCl (%, *w*/*v*)	0.5–1.0	0.5–2.0	0.5–1.0
Optimal growth pH	5.0–7.0	6.0–7.0	7.0–8.0
H_2_S production	+	+	−
Starch hydrolysis	−	+	+
Cellulose hydrolysis	−	+	+
Tween 20/40/60	−/+/+	+/+/+	−/+/−
API 50CH test			
L-Arabinose	−	+	+
Mannose	−	−	+
D-Turanose	−	w	−
Assimilation of:			
D-Maltose	−	+	+
D-Raffinose	−	−	+
L-Aspartic acid	−	+	−
Fusidic acid	+	−	−
D-Galacturonic acid	+	−	+
L-Glutamic acid	+	+	−
Polar lipids	DPG, PG, PE, PL	ND	L, DPG, PG, PE, APL, PL
Major fatty acids (>10%)	anteiso-C_15:0_, C_16:0_	anteiso-C_15:0_, C_16:0_	anteiso-C_15:0_, iso-C_16:0_, iso-C_14:0_
DNA G + C content (%)	50.5	44.3	44.0

Note: 1, strain ES5-4^T^; 2, *Paenibacillus dongdonensis* KCTC 33221^T^; 3, *Paenibacillus oceanisediminis* JCM 17814^T^. DPG, diphosphatidylglycerol; PG, phosphatidylglycerol; PE, phosphatidylethanolamine; PL, unidentified phospholipid; L, unidentified lipid; APL, unidentified aminophospholipid. +, positive; w, weakly positive; −, negative; ND, not determined. Data for taxa 1–3 were collected at the same time during the study.

## Data Availability

The data supporting the conclusions of this article are included within the article and its additional files. The 16S rRNA gene sequence of strain ES5-4^T^ was deposited at NCBI database with the accession number of OR584159. The whole-genome sequence of strain ES5-4^T^ was deposited at GenBank/EMBL/DDBJ with the accession number of JAVYAE000000000.

## Supplementary Materials

The following supporting information can be downloaded at https://www.mdpi.com/article/10.3390/microorganisms13071559/s1. Figure S1: Maximum-parsimony phylogenetic tree based on 16S rRNA gene sequences showing the position of the ES5-4T strain. Bootstrap values (expressed as percentages of 1000 replications) of >70 (%) are shown at the branch nodes. *Bacillus subtilis* NCIB 3610T is used as the outgroup. Figure S2: Neighbor-joining phylogenetic tree based on 16S rRNA gene sequences showing the position of the ES5-4T strain. Bootstrap values (expressed as percentages of 1000 replications) of >70 (%) are shown at the branch nodes. *Bacillus subtilis* NCIB 3610T is used as the outgroup. Figure S3: Effects of Se4+ concentration (20–5000 mg/L) on the growth of the ES5-4T strain cultured in TSB media at 28 °C for 2 days. Figure S4: Effects of Se4+ concentration (20–5000 mg/L) on the growth of the ES5-4T strain cultured on TSA media at 28 °C for 2 days. Figure S5: A two-dimensional thin-layer chromatogram of polar lipids. Note: a, *Paenibacillus enshiensis* ES5-4T; b, *Paenibacillus oceanisediminis* JCM17814T. DPG, diphosphatidylglycerol; PG, phosphatidylglycerol; PE, phosphatidylethanolamine; PL, unidentified phospholipid; L, unidentified lipid; APL, unidentified aminophospholipid. Table S1: Cellular fatty acid profiles (% of the total) of the ES5-4T strain and its closely related type strains of the genus *Paenibacillus*. Dataset S1: Annotated selenocompound metabolism gene profiles for the genus *Paenibacillus*.

## Abbreviations

The following abbreviations are used in this manuscript:

ANI	Average nucleotide identity
ANIb	Average nucleotide identity calculated by the alignment algorithms BLAST+
APL	Unidentified aminophospholipid
bp	Base pairs
COG	Cluster of Orthologous Groups
dDDH	The digital DNA–DNA hybridization
DPG	Diphosphatidylglycerol
GGDC	Genome-to-Genome Distance Calculator
GO	Gene Ontology
ICNP	International Code of Nomenclature of Prokaryotes
KEGG	Kyoto Encyclopedia of Genes and Genomes
L	Unidentified lipid
MCCC	Marine Culture Collection of China
ME	Minimum evolution
MIDI	Microbial identification system
ML	Maximum likelihood
NCBI	National Center of Biotechnology Information
NJ	Neighbor-joining
PCR	Polymerase chain reaction
PE	Phosphatidylethanolamine
PG	Phosphatidylglycerol
PGPB	Plant growth-promoting bacteria
PGPR	Plant growth-promoting rhizobacteria
PL	Unidentified phospholipid
Se	Selenium
SEM	Scanning electron microscopy
sp. nov	Species novum
TEM	Transmission electron microscopy
TSA	Trypticase Soy Agar
TSB	Trypticase soy broth
