# Supplementary material for: Paenibacillus hubeiensis sp. nov.: A Novel Selenium-Resistant Bacterium Isolated from the Rhizosphere of Galinsoga parviflora in a Selenium-Rich Region of Enshi, Hubei Province
Source: Microorganisms. 2025 Jul 2;13(7):1559. doi: 10.3390/microorganisms13071559 (PMC12300063; doi:10.3390/microorganisms13071559)
Supplement: Supplementary file 1 [file microorganisms-13-01559-s001.zip › Revised Supplementary Material.pdf]

Table S1. Cellular fatty acid profiles (% of the total) of the ES5-4^T strain and its closely related type strains of the genus *Paenibacillus*.

Fatty acid	1	2	3
Saturated			
$C_{14:0}$	2.1	1.8	5.9
$C_{15:0}$	1.7	6.6	4.4
$C_{16:0}$	**21.7**	7.8	**18.3**
$C_{17:0}$	TR	TR	TR
$C_{18:0}$	1.5	TR	TR
Unsaturated			
$C_{15:1}\omega 5c$	5.5	ND	ND
$C_{16:1}\omega 11c$	0.8	0.5	1.5
$C_{18:1}\omega 9c$	ND	TR	ND
Branched			
anteiso-$C_{13:0}$	ND	TR	ND
iso-$C_{14:0}$	4.4	**10.1**	4.7
iso-$C_{15:0}$	1.8	3.0	6.1
anteiso-$C_{15:0}$	**46.5**	**45.6**	**50.3**
iso-$C_{16:0}$	4.1	**20.0**	4.6
iso-$C_{17:0}$	TR	0.9	1.2
anteiso-$C_{17:0}$	TR	2.6	1.8
Hydroxy			
$C_{12:0}$2OH	4.5	ND	ND
$C_{12:0}$3OH	4.1	ND	ND

Note: 1, strain ES5-4^T (data from this study); 2, *P. oceanisediminis* JCM 17814^T (Lee et al. 2013); 3, *P. dongdonensis* KCTC 33221^T (Son et al. 2014). Fatty acids, which exceed 0.5% of the total fatty acids, are listed; The major fatty acids (>10%) values are in bold; TR: trace amount (< 0.5%); ND: not detected.

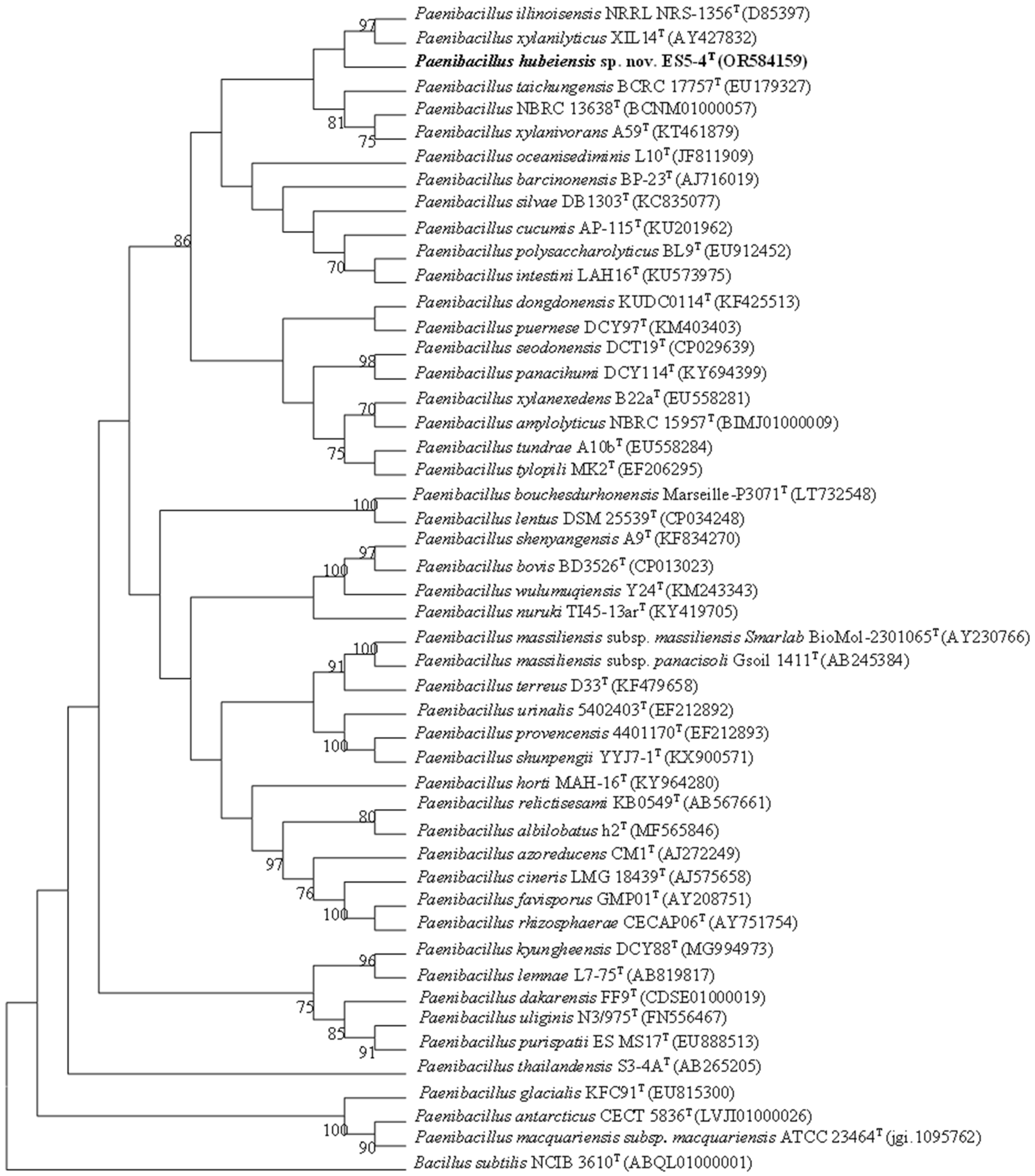

Figure S1. Maximum-parsimony phylogenetic tree based on 16S rRNA gene sequences showing the position of the ES5-4^T strain. Bootstrap values (expressed as percentages of 1,000 replications) of > 70 (%) are shown at the branch nodes. *Bacillus subtilis* NCIB 3610^T is used as the out group.

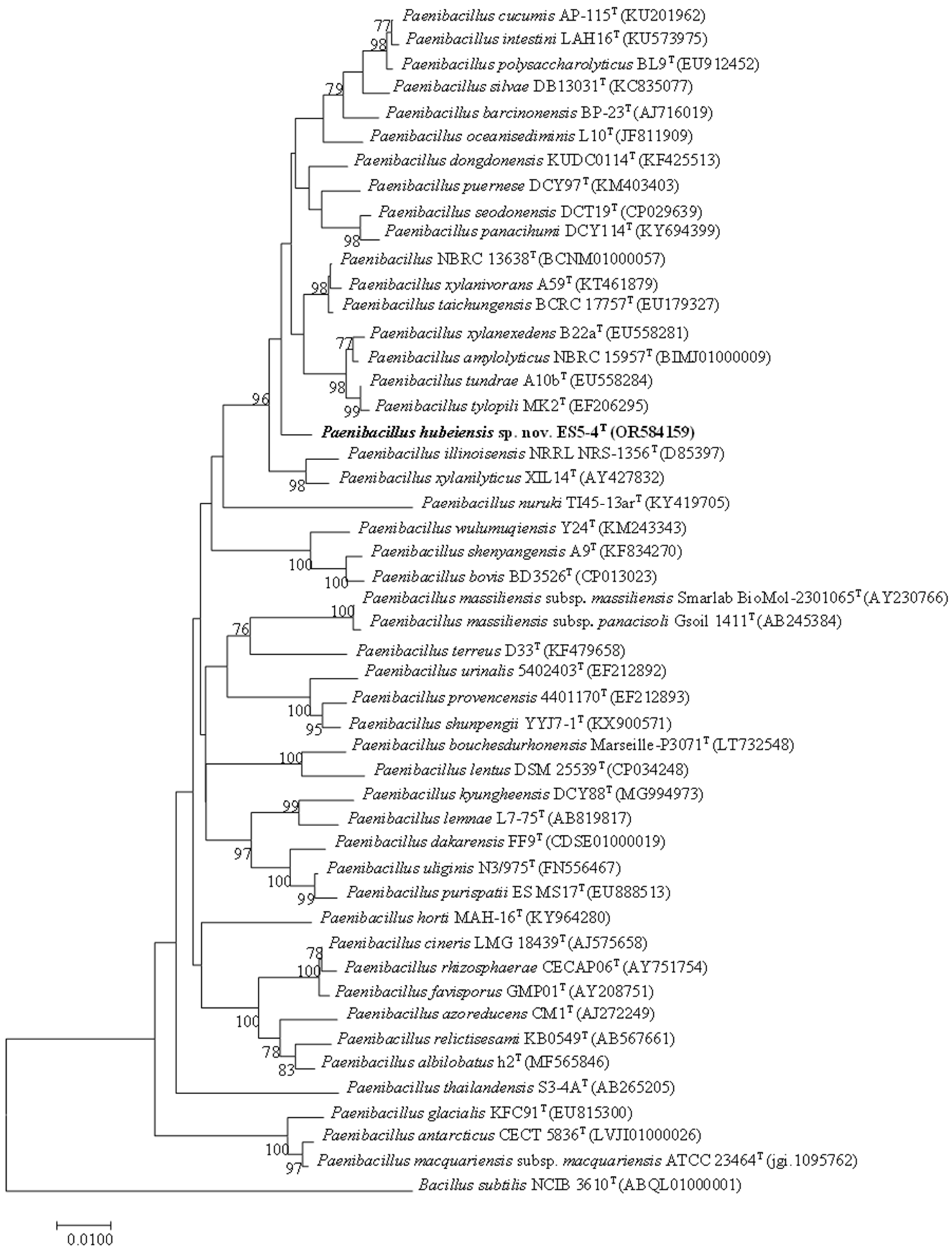

Figure S2. Neighbor-joining phylogenetic tree based on 16S rRNA gene sequences showing the position of the ES5-4^T strain. Bootstrap values (expressed as percentages of 1,000 replications) of > 70 (%) are shown at the branch nodes. *Bacillus subtilis* NCIB 3610^T is used as the out group.

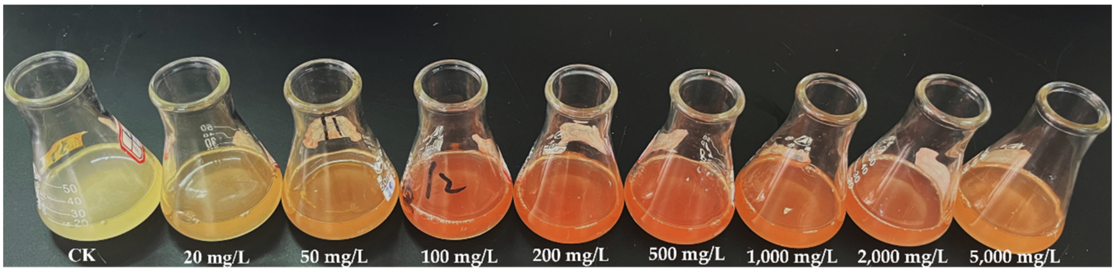

Figure S3. Effects of Se^{4+} concentration (20–5,000 mg/L) on the growth of the ES5-4^{T} strain cultured in TSB media at 28 °C for 2 days.

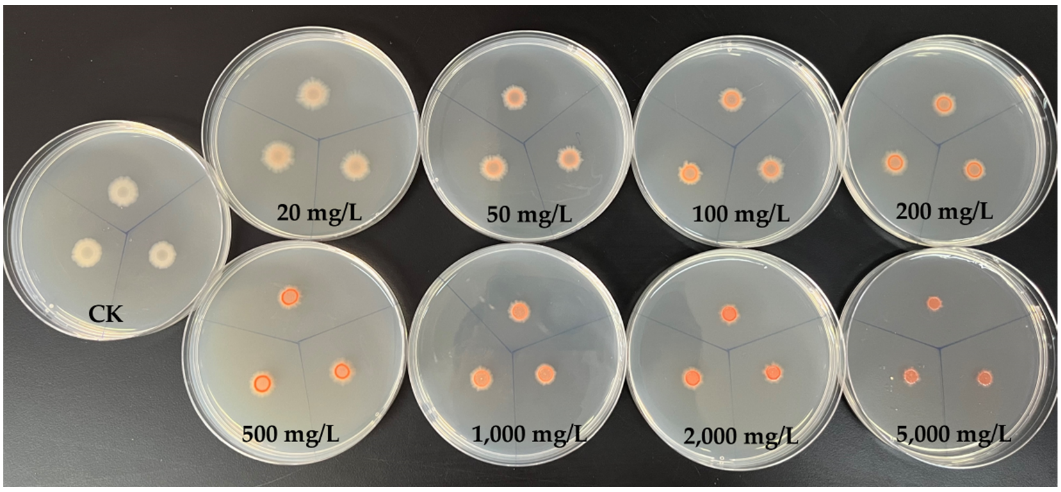

Figure S4. Effects of Se^{4+} concentration (20–5,000 mg/L) on the growth of the ES5-4^{T} strain cultured on TSA media at 28 °C for 2 days.

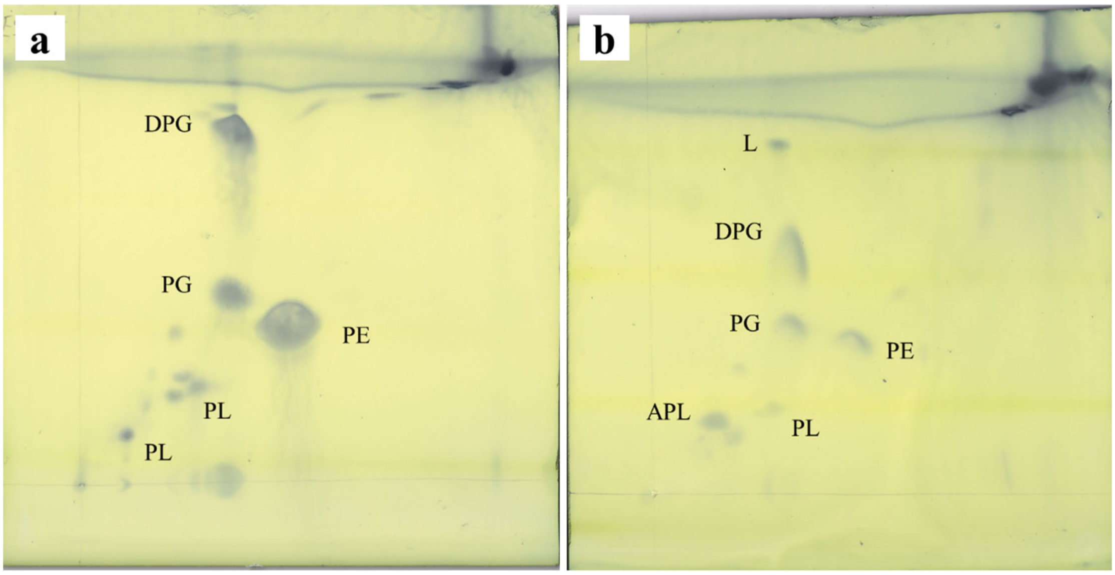

Figure S5. A two-dimensional thin-layer chromatogram of polar lipids

Note: a, *Paenibacillus enshiensis* ES5-4^{T}; b, *Paenibacillus oceanisediminis* JCM17814^{T}.

DPG, diphosphatidylglycerol; PG, phosphatidylglycerol; PE, phosphatidylethanolamine; PL, unidentified phospholipid; L, unidentified lipid; APL, unidentified aminophospholipid
